# Comparison of the measurement properties of SF-6Dv2 and EQ-5D-5L in a Chinese population health survey

**DOI:** 10.1186/s12955-022-02003-y

**Published:** 2022-06-16

**Authors:** Shitong Xie, Dingyao Wang, Jing Wu, Chunyu Liu, Wenchen Jiang

**Affiliations:** 1 Department of Health Research Methods, Evidence, and Impact, McMaster University, Hamilton, ON Canada; 2 School of Pharmaceutical Science and Technology, Tianjin University, Room 209, 24th Building, 92th Weijin Road, Nankai District, Tianjin, 300072 China; 3 Center for Social Science Survey and Data, Tianjin University, Tianjin, China; 4 Tianjin Health Information Research Center (Tianjin Health Development Research Center), Tianjin, China; 5 Tianjin Hospital of Integrated Traditional Chinese and Western Medicine, 6th Changjiang Road, Nankai District, Tianjin, 300100 China

**Keywords:** Health-related quality of life, SF-6Dv2, EQ-5D-5L, Measurement properties, Population health survey

## Abstract

**Background:**

SF-6Dv2, the latest version of SF-6D, has been developed recently, and its measurement properties remain to be evaluated and compared with the EQ-5D-5L. The aim of this study was to assess and compare the measurement properties of the SF-6Dv2 and the EQ-5D-5L in a large-sample health survey among the Chinese population.

**Methods:**

Data were obtained from the 2020 Health Service Survey in Tianjin, China. Respondents were randomly selected and invited to complete both the EQ-5D-5L and SF-6Dv2 through face-to-face interviews or self-administration. Health utility values were calculated by the Chinese value sets for the two measures. Ceiling and floor effects were firstly evaluated. Convergent validity and discriminate validity were examined using Spearman’s rank correlation and effect sizes, respectively. The agreement was assessed using intraclass correlation coefficients (ICC). Sensitivity was compared using relative efficiency and receiver operating characteristic.

**Results:**

Among 19,177 respondents (49.3% male, mean age 55.2 years, ranged 18–102 years) included in this study, the mean utility was 0.939 (0.168) for EQ-5D-5L and 0.872 (0.184) for SF-6Dv2. A higher ceiling effect was observed in EQ-5D-5L than in SF-6Dv2 (72.8% vs. 36.1%). The Spearman’s rank correlation (range: 0.30–0.69) indicated an acceptable convergent validity between the dimensions of EQ-5D-5L and SF-6Dv2. The SF-6Dv2 showed slightly better discriminative capacities than the EQ-5D-5L (ES: 0.126–2.675 vs. 0.061–2.256). The ICC between the EQ-5D-5L and SF-6Dv2 utility values of the total sample was 0.780 (*p* < 0.05). The SF-6Dv2 had 29.0–179.2% higher efficiency than the EQ-5D-5L at distinguishing between respondents with different external health indicators, while the EQ-5D-5L was found to be 8.2% more efficient at detecting differences in self-reported health status than the SF-6Dv2.

**Conclusions:**

Both the SF-6Dv2 and EQ-5D-5L have been demonstrated to be comparably valid and sensitive when used in Chinese population health surveys. The two measures may not be interchangeable given the moderate ICC and the systematic difference in utility values between the SF-6Dv2 and EQ-5D-5L. Further research is warranted to compare the test–retest reliability and responsiveness.

**Supplementary Information:**

The online version contains supplementary material available at 10.1186/s12955-022-02003-y.

## Introduction

Health-related quality of life (HRQoL) has been extensively used worldwide as a multidimensional concept that could be used to assess an individual’s health status based on physical, mental, and social functioning [1–3]. HRQoL can be evaluated using generic preference-based measures (GPBMs), which are commonly used in economic evaluations of healthcare interventions [4, 5]. A GPBM consists of a health state descriptive system and a corresponding country-specific health utility value set elicited from a representative sample of the general population. The health utility lies on a standard scale, where the upper boundary 1 refers to full health, 0 refers to death, and values lower than 0 refer to the health states that are deemed as worse than death. It provides a standardized weight to interpret the severity of the health state [6]. Given the acceptable cognitive burden for the respondents, the GPBMs are increasingly used in population health surveys [7–9]. A population health survey provides integral information on the overall situation and longitudinal trend of the health status of the residents, as well as the empirical evidence for supporting healthcare decision-making [10, 11].

The EQ-5D and the Short Form Six-Dimension (SF-6D) are the two most frequently used GPBMs worldwide [12, 13]. The EQ-5D was developed by the EuroQol Group, and currently has two versions, i.e., the EQ-5D-3L and the EQ-5D-5L. Both versions have the same dimensions to describe health states, while having different response levels (three levels in EQ-5D-3L and five levels in EQ-5D-5L) for each dimension [14, 15]. In comparison with the EQ-5D-3L, the EQ-5D-5L defines a wider range of health state descriptions, thus reducing ceiling effects and enhancing discriminant properties [15–17]. The original version of the SF-6D (SF-6Dv1) was developed based on the 36-item Short-Form Health Survey (SF-36) in 2002 and comprises six dimensions [18]. These dimensions are combined with four to six levels of severity, yielding up to 18,000 health states [18]. Another version of the SF-6D was developed based on the 12-item Short-Form Health Survey (SF-12) in 2004 [19]. It has the same six dimensions but different levels in each (three to five levels), defining 7500 health states [19]. More detailed information and empirical evidence of the difference between these two versions can be found elsewhere [9, 18, 19]. The newest version of the SF-6D, the SF-6Dv2, was recently developed by revising the ambiguity between the dimension levels and unifying the inconsistency of positive and negative wording in the SF-6Dv1 [20, 21].

Several studies have been conducted to compare the measurement properties of the EQ-5D and SF-6D in various types of diseases, such as diabetes, cardiovascular disease, cancer, chronic obstructive pulmonary disease, and end-stage renal disease [22–41]. All these studies were conducted to compare the SF-6Dv1 with EQ-5D-3L or EQ-5D-5L. A common finding in most studies was that the EQ-5D and SF-6D appeared to be generally reliable, valid, and responsive (or sensitive) to measure the HRQoL among the disease populations. Although the test–retest reliability of the SF-6D might be higher than that of the EQ-5D [33], the results of comparing discriminate validity (or known-group validity), as well as the responsiveness, were not consistent across studies [22, 23, 25–27, 29–33, 38, 40, 41].

Nevertheless, there have been only a few comparisons between the EQ-5D and SF-6D among the general population or in population health surveys [11, 42–46]. Most of these studies involved the EQ-5D-3L and SF-6Dv1, except one study which was conducted to compare the EQ-5D-5L with SF-6D (derived from the SF-12) in the Thai general population [42]. Although generally good convergent validity between the EQ-5D and SF-6D was observed [42–44], the discriminate validity varied across different studies. For example, Zhao et al. [43] found that the SF-6Dv1 had a higher level of discriminant validity than the EQ-5D-3L, while Bharmal et al. [45] illustrated that the EQ-5D-3L performed better than the SF-6Dv1 in the discriminative power. The responsiveness was compared in only one study, and it was found that the EQ-5D-5L was more responsive than the SF-6D (derived from the SF-12) for the respondents with worse health status [42]. No studies have been conducted to compare the reliability of the EQ-5D and SF-6D in the general population. Therefore, evidence comparing the measurement properties of the SF-6Dv2 and EQ-5D in the general population, especially in population health surveys, is still lacking worldwide.

Given that the SF-6Dv2 has been used in various countries [47, 48], and the Chinese version of SF-6Dv2 and its corresponding utility value set has been developed recently [49, 50], its measurement properties remain to be evaluated and compared with the EQ-5D-5L. Therefore, the aim of this study was to assess and compare the measurement properties of the SF-6Dv2 and EQ-5D-5L in a large-sample health survey among the Chinese population.

## Methods

### Data source

Data used in the study were obtained from the 2020 Tianjin Health Service Survey, which was conducted by Tianjin Health Commission between July and August 2020 [51]. Tianjin is one of the four municipalities of China, with a total of 16 districts and more than 15 million permanent population [52]. A multi-stage, stratified cluster random sampling strategy was used. First, five subdistricts (or townships) in each of the 16 districts were randomly selected. Second, two communities (or villages) were randomly selected within each of the 80 subdistricts (or townships). Third, 60 households were randomly selected within each of the 160 communities (or villages), and consequently, a total of 9600 households were included. All residents registered under each household were invited to participate in the survey.

Data from the 2020 Tianjin Health Service Survey were collected through three different approaches in this study to comply with the COVID-19 administrative policy in China, including face-to-face paper-based interviews at resident’s home, face-to-face paper-based interviews in publicly unified places (governmental subdistrict office or community health service center), and self-report at resident’s home. The process of the face-to-face interview was as follows. First, the respondent who was the most familiar with their family situations answered the basic questions, including the annual household medication expenditure and the distance to the closest healthcare institute from home. Second, all respondents provided a series of demographic characteristics (e.g., gender and age) and socioeconomic status (e.g., education level, marital and employment status). Third, respondents aged ≥ 15 years completed both the EQ-5D-5L and SF-6Dv2, then answered health indicator questions, including the presence of chronic diseases, presence of health examinations, and presence of illnesses in the last two weeks. Forth, questions referring to children aged < 5 years and including the number of health examinations within the past twelve months and the presence of vaccination certificates were posed to their parents. Fifth, female respondents aged 15–64 years were asked questions about the number of their children and the delivery place. Last, all respondents were asked about their knowledge and satisfaction with the hierarchical diagnosis and treatment model developed in China. Informed consent was obtained from all respondents included in the survey. Detailed information on sampling and data collection can be found elsewhere [51].

For this study, data collected in the second and third parts of the survey were used. Respondents aged < 18 years were excluded from this study since both the EQ-5D-5L and SF-6Dv2 are recommended to be used among adult respondents [20, 53]. Respondents were also required to meet the following inclusion criteria: (1) had no missing data for the EQ-5D-5L and SF-6Dv2 measures; and (2) had no missing data for the variables used in this study, including demographic characteristics, socioeconomic status, and health indicators.

### Measures

#### EQ-5D-5L

The EQ-5D-5L descriptive system comprises five dimensions, namely, mobility, self-care, usual activities, pain/discomfort, and anxiety/depression, each with five levels of severity (no, slight, moderate, severe, and extreme problems). A visual analog scale (hereafter EQ VAS) using a scale ranging from 0 (worst imaginable health state) to 100 (best imaginable health state) is also included in the EQ-5D-5L [15]. The EQ-5D-5L defines 3125 (= 5^5^) different health states according to all the possible combinations of dimension levels. The Chinese EQ-5D-5L utility value set was developed using the time trade-off (TTO) approach, with the range of − 0.391 (55,555) to 1 (11,111) [54].

#### SF-6Dv2

The SF-6Dv2 is derived from 10 items of the SF-36. The health state classification system of SF-6Dv2 comprises six dimensions, including physical functioning, role limitation, social functioning, pain, mental health, and vitality. The pain dimension has six response levels, while all others have five levels, resulting in 18,750 (= 5*5*5*6*5*5) different health states [20]. The Chinese SF-6Dv2 value set was developed using the TTO approach, with the range of − 0.277 (555,655) to 1 (111,111) [49].

### Statistical analysis

#### Descriptive statistics

The characteristics of respondents were described using means and standard deviations (SD) for continuous variables and frequencies and proportions for categorical variables. The distribution of response levels on each dimension of EQ-5D-5L and SF-6Dv2 was reported using histograms. Descriptive statistics (mean, SD) for the EQ-5D-5L and SF-6Dv2 utility values, and the EQ VAS scores were also computed. The EQ VAS scores were adopted as an indicator of self-reported health status, which was classified into four sub-groups: < 65 (bad), 65–79 (fair), 80–89 (good), and 90–100 (excellent) in this study [27, 41, 55].

#### Agreement

The agreement between EQ-5D-5L and SF-6Dv2 was examined using the intraclass correlation coefficient (ICC), which was computed with the two-way mixed-effects model based on absolute agreement [56]. An ICC above 0.7 suggests an acceptable agreement [57]. Besides, given that the distributions of utility values were highly skewed, the paired comparisons between the EQ-5D-5L and SF-6Dv2 utility values were examined using Wilcoxon signed-rank test [34].

#### Measurement properties of the EQ-5D-5L and SF-6Dv2

The measurement properties evaluated in this study included the ceiling and floor effects, convergent validity, discriminate validity, agreement, and sensitivity of the EQ-5D-5L and SF-6Dv2.

##### Ceiling and floor effects

Ceiling and floor and effects for each measure were assessed by examining the percentage of respondents in the best and worst health states, respectively. These effects are considered existing if more than 15% of the respondents achieved either extreme end of the scale [58].

##### Convergent validity

Convergent validity refers to the extent to which an outcome of interest (such as the pain/discomfort dimension in EQ-5D-5L) shows an expected association with another similar outcome (such as the pain dimension in SF-6Dv2) measured at the same time point [30, 59]. Convergent validity was assessed by examining the correlation between EQ-5D-5L and SF-6Dv2 dimensions using Spearman’s rank correlation coefficient (r). An absolute coefficient value greater than 0.5 stands for a strong correlation, values between 0.35–0.49 for moderate, values between 0.2 and 0.34 for weak, and values smaller than 0.2 for poor correlation [28, 60].

##### Discriminate validity

The mean utility value of each measure was calculated and compared to evaluate the capacity to discriminate between each of the respondents’ characteristic groups. The t-tests for dichotomous variables (e.g., gender) and the one-way analyses of variance for polytomous variables (e.g., age group and body mass index [BMI] group) were used, respectively. Effect sizes (ES) were also used to define the discriminative capacity of the EQ-5D-5L and SF-6Dv2, which was calculated as the difference between the mean utility of two sub-groups divided by the pooled standard deviation [61]. For polytomous variables, the effect sizes between the extreme sub-groups (e.g., the effect sizes between the aged 18–29 sub-group and the aged ≥ 70 sub-group) were calculated [11]. The larger effect size indicates the better discriminative ability of the measures [11, 34, 36, 42, 62]. As an extended test of validity, known-group validity was used to assess the extent to which an outcome measure of interest helps distinguish between subgroups that are theoretically expected to differ [30]. Based on the published literature [34, 42, 44], we hypothesized that the elder, the female, and the obese respondents, as well as respondents with poorer self-reported health status and chronic diseases, such as hypertension and diabetes, had lower utility values.

##### Sensitivity

The sensitivity of EQ-5D-5L and SF-6Dv2 for detecting differences in both external and self-reported health indicators were tested using the relative efficiency (RE) statistic. RE was determined via the ratio of the square of t-statistics from the t-tests of the comparator measure (SF-6Dv2) over that of the reference measure (EQ-5D-5L) [42, 43, 46]. A RE value of 1.0 indicates that the SF-6Dv2 has the same efficiency as EQ-5D-5L at detecting differences in these external health indicators. A value higher than 1 indicates that the SF-6Dv2 is more sensitive than the EQ-5D-5L, while a value lower than 1 means the opposite [63]. The receiver operating characteristic (ROC) curve was also used to evaluate the sensitivity of these two measures. The ROC curve provides a useful method to assess the performance of measures against external dichotomous variables of health status [64]. The area under the ROC curve (AUC) was computed to compare the discriminative power of the EQ-5D-5L and SF-6Dv2 [65]. The one that generates the larger AUC is regarded as more sensitive or effective at detecting differences, and measures with excellent discriminative ability would have an AUC score of 1.0, whereas an AUC score of 0.5 means no discriminative capacity [63]. For the current analyses, the presence of chronic diseases (i.e., hypertension and diabetes), illnesses in 2 weeks, and hospitalizations in 12 months represented the external health indicators. The self-reported health status was dichotomized as (1) excellent versus good, fair, or bad, (2) excellent or good versus fair or bad, and (3) excellent, good, or fair versus bad.

The statistical analyses were performed using STATA 15.0 (StataCorp LLC, College Station, TX, USA). All reported statistical tests were performed two-sided with a significance level of 0.05.

## Results

### Descriptive statistics

**Fig. 1:**
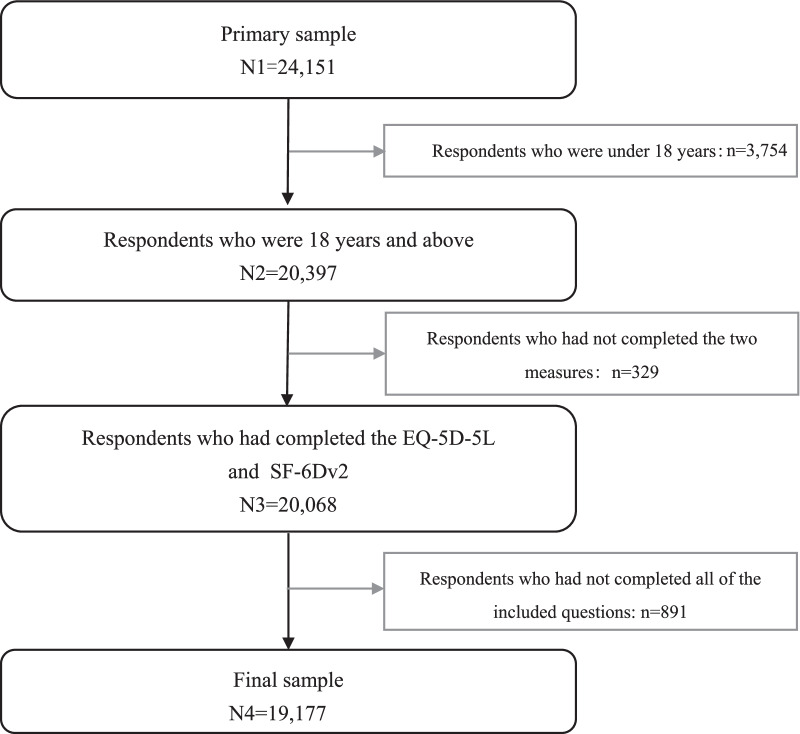
The flowchart of the sample inclusion for the comparison study

Of 24,151 respondents who participated in the survey, 4974 respondents were excluded from the current analyses because they were under 18 years (N = 3754), had not completed the EQ-5D-5L or SF-6Dv2 (N = 329), or had missing values among questions included in this study (N = 891). Finally, a total of 19,177 respondents were included (Fig. 1). As shown in Table 1, 49.3% (N = 9453) of respondents were male, and the mean (SD) age was 55.2 (16.2) years, with a range from 18 to 102 years. 35.5% (N = 6806) and 13.5% (N = 2586) of respondents had hypertension and diabetes, respectively.

**Table 1:** Characteristics of respondents and EQ-5D-5L and SF-6Dv2 utility values (N = 19,177)

Characteristics	Population N (%)
*Gender*	
Male	9453 (49.3%)
Female	9724 (50.7%)
*Ethnic group*	
Han Chinese	18,862 (98.4%)
Others	315 (1.6%)
*Age (mean[SD])*	55.2 (16.2)
*Age group (years)*	
18–29	1655 (8.6%)
30–39	2319 (12.1%)
40–49	2317 (12.1%)
50–59	3615 (18.9%)
60–69	5830 (30.4%)
≥ 70	3441 (17.9%)
*BMI*^*a*^* (mean[SD])*	24.4 (3.5)
*BMI group*^*b*^	
< 18.5 (thin)	551 (2.9%)
18.5–24 (normal)	8549 (44.6%)
24–28 (overweight)	7470 (38.9%)
≥ 28 (obese)	2607 (13.6%)
*Basic medical insurance*	
Urban employee	9394 (49.0%)
Urban and rural resident	9447 (49.3%)
No	336 (1.7%)
*Recipients of medical assistance*	
Yes	391 (2.0%)
No	18,786 (98.0%)
*Marital status*	
Unmarried	1736 (9.1%)
Married	15,833 (82.6%)
Widowed	1285 (6.7%)
Divorced	323 (1.6%)
*Education*	
Primary or below	4385 (22.9%)
Junior high school	7365 (38.4%)
Senior high school	3923 (20.5%)
College or above	3504 (18.2%)
*Employment status*	
Employed	7035 (36.7%)
Retired	6279 (32.7%)
Student	429 (2.2%)
Unemployed	5434 (28.4%)
*Health records*	
Yes	15,545 (81.1%)
No	3632 (18.9%)
*Health examination*	
Yes	11,964 (62.4%)
No	7213 (37.6%)
*Hypertension*	
Yes	6806 (35.5%)
No	12,371 (64.5%)
*Diabetes*	
Yes	2586 (13.5%)
No	16,591 (86.5%)
*Other chronic diseases*	
Yes	1082 (5.6%)
No	18,095 (94.4%)
*Number of illnesses in 2 weeks*	
0	17,523 (91.4%)
1	1377 (7.2%)
2 or more	277 (1.4%)
*Hospitalizations in 12 months*	
Yes	733 (3.8%)
No	18,444 (96.2%)
*EQ-5D-5L utility (mean [SD])*	0.939 (0.168)
*SF-6Dv2 utility (mean [SD])*	0.872 (0.184)
*EQ VAS score (mean [SD])*	84.4 (14.0)
*EQ VAS group*	
≥ 90 (excellent)	10,243 (53.4%)
80–89 (good)	5037 (26.3%)
65–79 (fair)	2217 (11.5%)
< 65 (bad)	1680 (8.8%)

*SD* Standard deviation

^a^*BMI* body mass index, equals weight(kg) divided by height(m) squared

^b^BMI groups were defined according to the guideline published by the Cooperative Meta-analysis Group of China Obesity Task Force in 2002 [66]

**Fig. 2:**
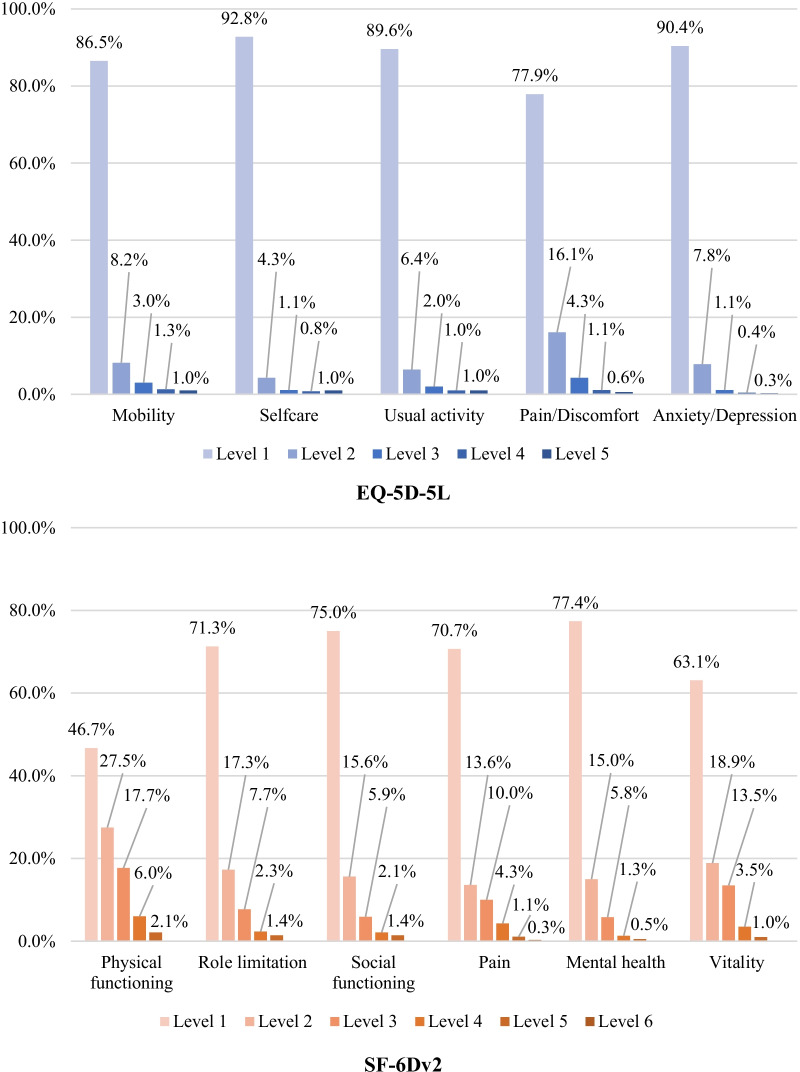
The distribution across levels of the EQ-5D-5L and SF-6Dv2 dimensions (N = 19,177). *Note*: Except for the pain dimension, which has six response levels, all others have five levels, with higher values representing more severe health states

The distribution of the responses to the EQ-5D-5L and SF-6Dv2 are presented in Fig. 2. An extreme majority of the respondents indicated no problems (level 1) on at least one of the five EQ-5D-5L dimensions, with the highest proportion appearing in self-care (92.8%), followed by anxiety/depression (90.4%), usual activities (89.6%), mobility (86.5%), and pain/discomfort (77.9%). Analogously, a large proportion of respondents were also classified in level 1 on the SF-6Dv2 dimensions of mental health (77.4%), followed by social functioning (75.0%), role limitation (71.3%), pain (70.7%), vitality (63.1%), and physical functioning (46.7%).

Of the total 19,177 respondents, the mean (SD) utility value of EQ-5D-5L was 0.939 (0.168), while that of SF-6Dv2 was 0.872 (0.184). The mean (SD) score of EQ VAS was 84.4 (14.0) (Table 1).

### Agreement

The ICC between the EQ-5D-5L and SF-6Dv2 utility values of the total sample was 0.780 (*p* < 0.05). Besides, the SF-6Dv2 utility values were significantly lower than those of the EQ-5D-5L (*p* < 0.001).

### Measurement properties of the EQ-5D-5L and SF-6Dv2

#### Ceiling and floor effects

The proportion of respondents reporting the best state of EQ-5D-5L was 72.8% (N = 13,961), which showed strong ceiling effects, while only 0.2% (N = 35) of respondents reported the worst state. Similarly, 36.1% (N = 6921) of respondents reported the best state of SF-6Dv2, indicating a ceiling effect for the SF-6Dv2, while only 0.1% (N = 16) respondents reported the worst state.

#### Convergent validity

The dimensions of EQ-5D-5L and SF-6Dv2 were positively and moderately associated, with Spearman’s rank correlation coefficient ranging from 0.30 to 0.69 (*p* < 0.001). As expected, the EQ-5D-5L pain/discomfort dimension was strongly correlated with the SF-6Dv2 pain dimension (r = 0.69), and the EQ-5D-5L anxiety/depression dimension was highly correlated with the SF-6Dv2 mental health dimension (r = 0.52) (Additional file 1).

#### Discriminate validity

As reported in Table 2, both the EQ-5D-5L and SF-6Dv2 utility values were significantly different (*p* < 0.001) across groups defined by demographic characteristics, socioeconomic status, and health-related indicators, with effect sizes ranging from 0.061 to 2.256 for the EQ-5D-5L, and 0.126–2.675 for the SF-6Dv2. The effects sizes of the SF-6Dv2 were generally larger than the EQ-5D-5L. Moreover, the hypotheses for known-group validity were fulfilled in all tested groups (Table 2).

**Table 2:** Discriminative capacity and univariate analyses for EQ-5D-5L and SF-6Dv2 utility values within different groups (N = 19,177)

	EQ-5D-5L			SF-6D-v2		
	Mean (SD)	*p* Value	Effect size^a^ (95% CI)	Mean (SD)	*p* Value	Effect size^a^ (95% CI)
*Gender*		< 0.001	0.061 (0.032, 0.089)		< 0.001	0.126 (0.098, 0.154)
Male	0.944 (0.167)			0.884 (0.179)		
Female	0.934 (0.169)			0.860 (0.188)		
*Age group (years)*		< 0.001	0.719 (0.659, 0.780)		< 0.001	1.151 (1.088, 1.214)
18–29	0.994 (0.067)			0.977 (0.074)		
30–39	0.993 (0.050)			0.964 (0.081)		
40–49	0.984 (0.078)			0.939 (0.115)		
50–59	0.959 (0.129)			0.888 (0.159)		
60–69	0.934 (0.161)			0.846 (0.179)		
≥ 70	0.834 (0.267)			0.741 (0.245)		
*BMI*^*b*^* group*		< 0.001	0.258 (0.171, 0.344)		< 0.001	0.258 (0.171, 0.344)
< 18.5 (thin)	0.902 (0.249)			0.835 (0.252)		
18.5–24 (normal)	0.945 (0.160)			0.883 (0.179)		
24–28 (overweight)	0.942 (0.158)			0.872 (0.176)		
≥ 28 (obese)	0.920 (0.192)			0.844 (0.202)		
*Marital status*		< 0.001	0.723 (0.649, 0.798)		< 0.001	1.087 (1.010, 1.164)
Unmarried	0.975 (0.119)			0.950 (0.132)		
Married	0.943 (0.160)			0.873 (0.178)		
Widowed	0.835 (0.264)			0.745 (0.246)		
Divorced	0.959 (0.138)			0.894 (0.176)		
*Education*		< 0.001	0.515 (0.470, 0.560)		< 0.001	0.753 (0.707, 0.799)
Primary and below	0.880 (0.233)			0.787 (0.239)		
Junior high school	0.948 (0.150)			0.882 (0.164)		
Senior high school	0.954 (0.140)			0.890 (0.158)		
College or high	0.977 (0.103)			0.935 (0.127)		
*Employment status*		< 0.001	0.429 (0.330, 0.528)		< 0.001	0.694 (0.595, 0.793)
Employed	0.987 (0.069)			0.943 (0.105)		
Retired	0.918 (0.184)			0.833 (0.184)		
Student	0.988 (0.075)			0.971 (0.099)		
Unemployed	0.897 (0.220)			0.816 (0.230)		
*Health examination*		< 0.001	0.075 (0.046, 0.104)		< 0.001	0.236 (0.207, 0.265)
Yes	0.934 (0.166)			0.856 (0.184)		
No	0.947 (0.171)			0.899 (0.181)		
*Hypertension*		< 0.001	0.420 (0.390, 0.450)		< 0.001	0.600 (0.570, 0.630)
Yes	0.894 (0.216)			0.803 (0.216)		
No	0.963 (0.128)			0.909 (0.151)		
*Diabetes*		< 0.001	0.375 (0.333, 0.417)		< 0.001	0.502 (0.460, 0.544)
Yes	0.885 (0.224)			0.793 (0.220)		
No	0.947 (0.156)			0.884 (0.175)		
*Other chronic diseases*		< 0.001	0.830 (0.768, 0.892)		< 0.001	1.047 (0.984, 1.109)
Yes	0.810 (0.289)			0.695 (0.292)		
No	0.947 (0.154)			0.882 (0.170)		
*Number of illnesses in 2 weeks*		< 0.001	0.663 (0.544, 0.782)		< 0.001	0.932 (0.813, 1.051)
0	0.944 (0.160)			0.882 (0.174)		
1	0.891 (0.217)			0.779 (0.232)		
2 or more	0.837 (0.284)			0.717 (0.299)		
*Hospitalizations in 12 months*		< 0.001	0.851 (0.777, 0.926)		< 0.001	0.967 (0.893, 1.042)
Yes	0.803 (0.304)			0.703 (0.288)		
No	0.944 (0.158)			0.878 (0.176)		
*EQ VAS group*		< 0.001	2.256 (2.197, 2.315)		< 0.001	2.675 (2.613, 2.737)
≥ 90 (excellent)	0.989 (0.055)			0.946 (0.090)		
80–89 (good)	0.953 (0.108)			0.869 (0.136)		
65–79 (fair)	0.884 (0.184)			0.771 (0.177)		
< 65 (bad)	0.665 (0.358)			0.561 (0.312)		

T-tests were performed to identify statistically significant effects of dichotomous variables on utility values, while one-way analyses of variance were performed on polychromous variables

^a^The effect size was calculated as the difference between the mean utility of two sub-groups divided by the pooled standard deviation

^b^BMI: Body Mass Index, equals weight(kg) divided by height(m) squared. BMI groups were defined according to the guideline published by the Cooperative Meta-analysis Group of China Obesity Task Force in 2002 [66]

95% CI: 95% confidence interval; SD: standard deviation

#### Sensitivity

As shown in Table 3, the SF-6Dv2 was found to be 29.0–179.2% more efficient than the EQ-5D-5L at detecting differences in external health indicator groups, including hypertension, diabetes, other chronic diseases, illnesses in 2 weeks, and hospitalizations in 12 months. The SF-6Dv2 also had a 50.7–102.8% higher efficiency at revealing differences between self-reported health status groups dichotomized by “excellent” or “good” (Table 4). However, when the groups were dichotomized by “bad”, the EQ-5D-5L was found to be 8.2% more efficient at detecting the differences in self-reported health status (Table 4). The AUC values of both SF-6Dv2 and EQ-5D-5L were above 0.5 with statistically significant differences (*p* < 0.001) (Tables 3, 4). The SF-6Dv2 generated higher AUC scores than the EQ-5D-5L, indicating a possible sensitivity superiority.

**Table 3:** Sensitivity of EQ-5D-5L and SF-6Dv2 to detect differences in dichotomous health indicators (N = 19,177)

	Dichotomous health status groups	N	Utility value (Mean [SD])	t-test		RE^a^	ROC curve	
				t-statistic	*p* Value		AUC	95% CI
EQ-5D-5L	Hypertension	6806	0.894 (0.216)	− 27.812	< 0.001	1.000	0.626*	(0.619, 0.633)
	Non-hypertension	12,371	0.963 (0.128)					
SF-6Dv2	Hypertension	6806	0.803 (0.216)	− 39.772	< 0.001	2.045	0.699*	(0.692, 0.707)
	Non-hypertension	12,371	0.909 (0.151)					
EQ-5D-5L	Diabetes	2586	0.885 (0.224)	− 17.736	< 0.001	1.000	0.610*	(0.599, 0.620)
	Non-diabetes	16,591	0.947 (0.156)					
SF-6Dv2	Diabetes	2586	0.793 (0.220)	− 23.735	< 0.001	1.791	0.663*	(0.652, 0.674)
	Non-diabetes	16,591	0.884 (0.175)					
EQ-5D-5L	Other chronic diseases	1082	0.810 (0.289)	− 26.512	< 0.001	1.000	0.702*	(0.686, 0.718)
	Non-other chronic diseases	18,095	0.947 (0.154)					
SF-6Dv2	Other chronic diseases	1082	0.695 (0.292)	− 33.439	< 0.001	1.591	0.730*	(0.714, 0.746)
	Non-other chronic diseases	18,095	0.882 (0.170)					
EQ-5D-5L	Illnesses in 2 weeks	1654	0.882 (0.230)	− 14.448	< 0.001	1.000	0.605*	(0.592, 0.618)
	Non-illnesses in 2 weeks	17,523	0.944 (0.160)					
SF-6Dv2	Illnesses in 2 weeks	1654	0.769 (0.245)	− 24.141	< 0.001	2.792	0.676*	(0.663, 0.689)
	Non-illnesses in 2 weeks	17,523	0.882 (0.174)					
EQ-5D-5L	Hospitalizations in 12 months	733	0.803 (0.304)	− 22.606	< 0.001	1.000	0.683*	(0.663, 0.703)
	Non-hospitalizations in 12 months	18,444	0.944 (0.158)					
SF-6Dv2	Hospitalizations in 12 months	733	0.703 (0.288)	− 25.679	< 0.001	1.290	0.719*	(0.699, 0.738)
	Non-hospitalizations in 12 months	18,444	0.878 (0.176)					

*AUC* Area under the ROC curve, *95% CI* 95% confidence interval, *RE* Relative efficiency, *ROC* Receiver operating characteristic, *SD* Standard deviation

^a^RE of SF-6Dv2 is presented, and reference is EQ-5D-5L, of which RE is 1.000

*p < 0.001. For the ROC curve, *p* < 0.001 indicates that AUC is statistically significantly greater than 0.5 and that measure has discriminatory power

**Table 4:** Sensitivity of EQ-5D-5L and SF-6Dv2 to detect differences in dichotomous self-reported health status (N = 19,177)

	Dichotomous self-reported health status groups	N	Utility value (Mean [SD])	t-Test		RE^a^	ROC curve	
				t-Statistic	z-Statistic		AUC	95% CI
EQ-5D-5L	Excellent	10,243	0.989 (0.055)	− 46.513	< 0.001	1.000	0.698*	(0.692, 0.704)
	Good, fair or bad	8934	0.882 (0.226)					
SF-6Dv2	Excellent	10,243	0.946 (0.090)	− 66.232	< 0.001	2.028	0.780*	(0.773, 0.786)
	Good, fair or bad	8934	0.787 (0.223)					
EQ-5D-5L	Excellent or good	15,280	0.977 (0.078)	− 69.879	< 0.001	1.000	0.773*	(0.762, 0.781)
	Fair or bad	3897	0.789 (0.294)					
SF-6Dv2	Excellent or good	15,280	0.921 (0.113)	− 85.390	< 0.001	1.493	0.835*	(0.827, 0.842)
	Fair or bad	3897	0.680 (0.266)					
EQ-5D-5L	Excellent, good or fair	17,497	0.965 (0.103)	84.901	< 0.001	1.000	0.833*	(0.822, 0.844)
	Bad	1680	0.665 (0.358)					
SF-6Dv2	Excellent, good or fair	17,497	0.902 (0.133)	81.324	< 0.001	0.918	0.870*	(0.860, 0.879)
	Bad	1680	0.561 (0.312)					

*AUC* Area under the ROC curve, *95% CI* 95% confidence interval, *RE* Relative efficiency, *ROC* Receiver operating characteristic, *SD* Standard deviation

^a^RE of SF-6Dv2 is presented, and reference is EQ-5D-5L, of which RE is 1.000

*p < 0.001. For the ROC curve, *p* < 0.001 indicates that AUC is statistically significantly greater than 0.5 and that measure has discriminatory power

## Discussion

Both the EQ-5D and SF-6D have been widely applied in populations with specific diseases [22–41], while evidence on the comparison of their measurement properties in the general population is still lacking. To the best of our knowledge, this study provided the first evidence of comparing the measurement properties between the EQ-5D-5L and SF-6Dv2 in a large sample of the Chinese population.

While no floor effects were observed for either the EQ-5D-5L or SF-6Dv2 (0.2% vs. 0.1%), large ceiling effects (72.8% vs. 36.1%) were found for both measures. Previous studies conducted in the general population also yielded ceiling effects of approximately 43.3–73.6% for EQ-5D-3L [11, 43–46], and 49.1–54.0% for EQ-5D-5L [42, 67], while 1.0–18.3% for SF-6Dv1 [11, 43–46]. However, the ceiling effects found in this study were relatively higher than those in previous studies. One possible reason is that the Chinese population is more unwilling to report their health problems than the Western population due to the cultural tradition [68], which was confirmed by previous studies that the Chinese population reported higher ceiling effects than the Western populations [43, 44]. Another potential reason is that the respondents included in this study were in relatively better health status. Only 8.6% of them had experienced illnesses 2 weeks before the survey, which was much less than a study conducted among the general population in Chengdu city, China [43]. Moreover, the EQ-5D-5L showed a higher ceiling effect than the SF-6Dv2 in this study, which is consistent with previous studies where the EQ-5D-5L and SF-6D were compared in both general and disease populations [23, 27, 42]. This can be partly explained by the difference in the recall period, as the SF-6D frames its questions in terms of health “over the last 4 weeks”, while “today” is used in EQ-5D. A longer recall period may provide more scopes for respondents to include small impaired issues affecting their HRQoL that might not be detected during a relatively short period [69].

The ICC value between the EQ-5D-5L and SF-6Dv2 utility values indicated a moderate agreement (ICC = 0.780). This result is higher than those found in two previous studies. In one of the two studies, the ICC between the EQ-5D-5L and the SF-6D (derived from the SF-12) was 0.510 [42]. In the other study, the ICC between the EQ-5D-3L and SF-6Dv1 was 0.536 [44]. All findings reported above suggested that the SF-6Dv2 and EQ-5D-5L showed some similarities in detecting the trend of changes in health utility values, but might be different in the absolute amount of HRQoL measured. This could be partly explained by the different dimensions covered and the different utility ranges of the two measures (− 0.391 to 1 for EQ-5D-5L vs. − 0.227 to 1 for SF-6Dv2) [49, 54]. Therefore, the utility values of the SF-6Dv2 and EQ-5D-5L may not be interchangeable.

The correlation between the EQ-5D-5L and SF-6Dv2 dimensions (r = 0.30–0.69) was also acceptable, and better than the values in the previous study which the EQ-5D-3L and SF-6Dv1 were compared (r = 0.20–0.51) [43]. Both the EQ-5D-5L and SF-6Dv2 showed those utility differences between sociodemographic and health-related groups that were expected. However, these differences tended to be more apparent for the SF-6Dv2 with larger effects sizes (ES = 0.061–2.256 for EQ-5D-5L and 0.126–2.675 for SF-6Dv2). One of the possible reasons is that the SF-6Dv2 has one more dimension, resulting in a larger descriptive system than EQ-5D-5L (18,750 vs. 3125 health states). However, this result is different from the two previous studies. One study was conducted to compare the EQ-5D-5L with the SF-6D (derived from the SF-12) in the Thai general population (ES = 0.31–1.62 for EQ-5D-5L and 0.08–0.67 for SF-6D) [42]. The other study was conducted to compare the EQ-5D-3L with the SF-6Dv1 in the Spanish general population (ES = 0.17–1.33 for EQ-5D-3L and 0.14–1.33 for SF-6Dv1) [11]. An explanation of these contrasting findings might be that the SF-6Dv2 has revised the dimension levels and could describe more health states than the SF-6Dv1 or the SF-6D derived from the SF-12. Consequently, the known group validity of the SF-6Dv2 might be improved, which has been confirmed by the previous evidence [47].

Although both the SF-6Dv2 and EQ-5D-5L showed to be sensitive and efficient in this study, some merits of each measure are still worth to be emphasized. The SF-6Dv2 was more sensitive than the EQ-5D-5L to distinguish between different external health indicators. However, when it came to the dichotomous EQ VAS based self-reported health status groups, the sensitivity of the EQ-5D-5L and SF-6Dv2 varied in terms of the different choices of “cut-off” points. The EQ-5D-5L was more sensitive for differentiating between the self-reported health status with more impaired problems. These findings are inconsistent with two previous studies, which were conducted to compare the SF-6D with EQ-5D-3L and EQ-5D-5L, respectively [42, 46]. The AUC of SF-6Dv2 (0.663–0.870) was always higher than that of EQ-5D-5L (0.605–0.833) in all tested groups. This finding is similar to the study conducted in the US general population [46], but is contrary to another study carried out in the Spanish [11], both of which were compared the EQ-5D-3L with SF-6Dv1. Thus, which of the two measures is more sensitive remains unclear. Further studies are required to provide more evidence regarding this issue.

This study has several limitations. First, the respondents were recruited in one city and the average age of them was slightly high, which may have an impact on the representativeness of the general population in China. Second, both face-to-face interviews and self-reports were used to ask the respondents to complete the questionnaire, which may affect the validity of the results of this study to some extent. Third, given the main content of the health survey, i.e., the accessibility and satisfaction with the health services, the number of the external indicators of health status were limited in this study. Fourth, this study was conducted based on cross-sectional data instead of longitudinal data. Therefore, it was not possible to evaluate and compare the test–retest reliability and longitudinal responsiveness. Further investigations using longitudinal data are required to compare the test–retest reliability and responsiveness of the SF-6Dv2 and EQ-5D-5L.

## Conclusion

The SF-6Dv2 and EQ-5D-5L have been demonstrated to be comparably valid and sensitive when used in the Chinese population health survey. Given that the ICC value between the SF-6Dv2 and EQ-5D-5L is moderate and the utility values obtained from the two measures are systematically different, the SF-6Dv2 and EQ-5D-5L appear to be not interchangeable. Further research with a representative sample of the general population in China is needed to compare additional measurement properties of these two measures, such as test–retest reliability and longitudinal responsiveness.

## Supplementary Information

**Additional file 1: Table A1.** The convergent validity.

## Data Availability

Data are available from the authors upon reasonable request.

## Abbreviations

AUC: Area under the ROC curve
BMI: Body mass index
EQ-5D-3L: EQ-5D 3-level version
EQ-5D-5L: EQ-5D 5-level version
EQ VAS: EuroQol visual analog scale
ES: Effect sizes
GBPMs: Generic preference-based measures
HRQoL: Health-related quality of life
ICC: Intraclass correlation coefficient
RE: Relative efficiency
ROC: Receiver operating characteristic
SD: Standard deviations
SF-12: 12-Item Short-Form Health Survey
SF-36: 36-Item Short-Form Health Survey
SF-6D: Short Form Six-Dimension
TTO: Time trade-off

## References

[1] Hays RD, Reeve BB (2008). Measurement and modeling of health-related quality of life. Int Encycl Public Health.

[2] Wilson IB, Cleary PD (1995). Linking clinical variables with health-related quality of life. A conceptual model of patient outcomes. JAMA.

[3] Karimi M, Brazier J (2016). Health, health-related quality of life, and quality of life: what is the difference?. Pharmacoeconomics.

[4] Guyatt GH, Feeny DH, Patrick DL (1993). Measuring health-related quality of life. Ann Intern Med.

[5] Pynsent PB (2001). Choosing an outcome measure. J Bone Joint Surg Br.

[6] Neumann PJ, Sanders GD, Russell LB (2016). Cost-effectiveness in health and medicine.

[7] Hay JW, Gong CL, Jiao X (2021). A US population health survey on the impact of COVID-19 using the EQ-5D-5L. J Gen Intern Med.

[8] Sun S, Chen J, Kind P (2015). Experience-based VAS values for EQ-5D-3L health states in a national general population health survey in China. Qual Life Res.

[9] Luo N, Wang P, Fu AZ (2012). Preference-based SF-6D scores derived from the SF-36 and SF-12 have different discriminative power in a population health survey. Med Care.

[10] Macran S, Weatherly H, Kind P (2003). Measuring population health: a comparison of three generic health status measures. Med Care.

[11] Cunillera O, Tresserras R, Rajmil L (2010). Discriminative capacity of the EQ-5D, SF-6D, and SF-12 as measures of health status in population health survey. Qual Life Res.

[12] Brazier J, Ratcliffe J, Salomon JA (2017). Measuring and valuing health benefits for economic evaluation.

[13] The EuroQol Group (1990). EuroQol—a new facility for the measurement of health-related quality of life. Health Policy.

[14] Brooks R (1996). EuroQol: the current state of play. Health Policy.

[15] Herdman M, Gudex C, Lloyd A (2011). Development and preliminary testing of the new five-level version of EQ-5D (EQ-5D-5L). Qual Life Res.

[16] Thompson AJ, Turner AJ (2020). A comparison of the EQ-5D-3L and EQ-5D-5L. Pharmacoeconomics.

[17] Agborsangaya CB, Lahtinen M, Cooke T (2014). Comparing the EQ-5D 3L and 5L: measurement properties and association with chronic conditions and multimorbidity in the general population. Health Qual Life Outcomes.

[18] Brazier J, Roberts J, Deverill M (2002). The estimation of a preference-based measure of health from the SF-36. J Health Econ.

[19] Brazier J, Roberts J (2004). The estimation of a preference-based measure of health from the SF-12. Med Care.

[20] Brazier J, Mulhern BJ, Bjorner JB (2020). SF-6Dv2 International Project Group. Developing a new version of the SF-6D health state classification system from the SF-36v2: SF-6Dv2. Med Care.

[21] Poder TG, Fauteux V, He J (2019). Consistency between three different ways of administering the short form 6 dimension version 2. Value Health.

[22] Xu RH, Dong D, Luo N (2021). Evaluating the psychometric properties of the EQ-5D-5L and SF-6D among patients with haemophilia. Eur J Health Econ.

[23] Sun CY, Liu Y, Zhou LR (2021). Comparison of EuroQol-5D-3L and Short Form-6D utility scores in family caregivers of colorectal cancer patients: a cross-sectional survey in China. Front Public Health.

[24] Lamu AN, Björkman L, Hamre HJ (2021). Validity and responsiveness of EQ-5D-5L and SF-6D in patients with health complaints attributed to their amalgam fillings: a prospective cohort study of patients undergoing amalgam removal. Health Qual Life Outcomes.

[25] Selva-Sevilla C, Ferrara P, Gerónimo-Pardo M (2020). Interchangeability of the EQ-5D and the SF-6D, and comparison of their psychometric properties in a spinal postoperative Spanish population. Eur J Health Econ.

[26] Nikolova S, Hulme C, West R (2020). Normative estimates and agreement between 2 measures of health-related quality of life in older people with frailty: findings from the community ageing research 75+ cohort. Value Health.

[27] Ye Z, Sun L, Wang Q (2019). A head-to-head comparison of EQ-5D-5 L and SF-6D in Chinese patients with low back pain. Health Qual Life Outcomes.

[28] Thuppal S, Markwell S, Crabtree T (2019). Comparison between the EQ-5D-3L and the SF-6D quality of life (QOL) questionnaires in patients with chronic obstructive pulmonary disease (COPD) undergoing lung volume reduction surgery (LVRS). Qual Life Res.

[29] Kularatna S, Senanayake S, Gunawardena N (2019). Comparison of the EQ-5D 3L and the SF-6D (SF-36) contemporaneous utility scores in patients with chronic kidney disease in Sri Lanka: a cross-sectional survey. BMJ Open.

[30] Heslin M, Chua KC, Trevillion K (2019). Psychometric properties of the five-level EuroQoL-5 dimension and Short Form-6 dimension measures of health-related quality of life in a population of pregnant women with depression. BJPsych Open.

[31] Harvie HS, Honeycutt AA, Neuwahl SJ, et al; NICHD Pelvic Floor Disorders Network. Responsiveness and minimally important difference of SF-6D and EQ-5D utility scores for the treatment of pelvic organ prolapse. Am J Obstet Gynecol. 2019;220(3):265.e1–265.e11. 10.1016/j.ajog.2018.11.1094. 10.1016/j.ajog.2018.11.1094 PMC6401219 30471259

[32] Brown CC, Tilford JM, Payakachat N (2019). Measuring health spillover effects in caregivers of children with autism spectrum disorder: a comparison of the EQ-5D-3L and SF-6D. Pharmacoeconomics.

[33] Abdin E, Chong SA, Seow E (2019). A comparison of the reliability and validity of SF-6D, EQ-5D and HUI3 utility measures in patients with schizophrenia and patients with depression in Singapore. Psychiatry Res.

[34] Sayah FA, Qiu W, Xie F (2017). Comparative performance of the EQ-5D-5L and SF-6D index scores in adults with type 2 diabetes. Qual Life Res.

[35] Sakthong P, Munpan W (2017). A head-to-head comparison of UK SF-6D and Thai and UK EQ-5D-5L value sets in Thai patients with chronic diseases. Appl Health Econ Health Policy.

[36] Kularatna S, Byrnes J, Chan YK (2017). Comparison of the EQ-5D-3L and the SF-6D (SF-12) contemporaneous utility scores in patients with cardiovascular disease. Qual Life Res.

[37] Yousefi M, Najafi S, Ghaffari S (2016). Comparison of SF-6D and EQ-5D scores in patients with breast cancer. Iran Red Crescent Med J.

[38] Shah HA, Dritsaki M, Pink J (2016). Psychometric properties of patient reported outcome measures (PROMs) in patients diagnosed with acute respiratory distress syndrome (ARDS). Health Qual Life Outcomes.

[39] Yang F, Lau T, Lee E (2015). Comparison of the preference-based EQ-5D-5L and SF-6D in patients with end-stage renal disease (ESRD). Eur J Health Econ.

[40] Wu J, Han Y, Zhao FL (2014). Validation and comparison of EuroQoL-5 dimension (EQ-5D) and Short Form-6 dimension (SF-6D) among stable angina patients. Health Qual Life Outcomes.

[41] Zhao FL, Yue M, Yang H (2010). Validation and comparison of EuroQol and short form 6D in chronic prostatitis patients. Value Health.

[42] Kangwanrattanakul K (2021). A comparison of measurement properties between UK SF-6D and English EQ-5D-5L and Thai EQ-5D-5L value sets in general Thai population. Expert Rev Pharmacoecon Outcomes Res.

[43] Zhao L, Liu X, Liu D (2019). Comparison of the psychometric properties of the EQ-5D-3L and SF-6D in the general population of Chengdu city in China. Medicine (Baltimore).

[44] Kontodimopoulos N, Pappa E, Papadopoulos AA (2009). Comparing SF-6D and EQ-5D utilities across groups differing in health status. Qual Life Res.

[45] Bharmal M, Thomas J (2006). Comparing the EQ-5D and the SF-6D descriptive systems to assess their ceiling effects in the US general population. Value Health.

[46] Petrou S, Hockley C (2005). An investigation into the empirical validity of the EQ-5D and SF-6D based on hypothetical preferences in a general population. Health Econ.

[47] McDool E, Mukuria C, Brazier J (2021). A comparison of the SF-6Dv2 and SF-6D UK utility values in a mixed patient and healthy population. Pharmacoeconomics.

[48] Nahvijou A, Safari H, Ameri H (2021). Psychometric properties of the SF-6Dv2 in an Iranian breast cancer population. Breast Cancer.

[49] Wu J, Xie S, He X (2021). Valuation of SF-6Dv2 health states in China using time trade-off and discrete-choice experiment with a duration dimension. Pharmacoeconomics.

[50] Wu J, Xie S, He X (2020). The simplified Chinese version of SF-6Dv2: translation, cross-cultural adaptation and preliminary psychometric testing. Qual Life Res.

[51] Tianjin Health Commission: The 2020 Tianjin Health Service Survey. 2020. http://wsjk.tj.gov.cn. Accessed June 2020.

[52] National Bureau of Statistics of China. China Seventh National Census. 2020. http://stats.tj.gov.cn/tjsj_52032/tjgb/202105/t20210521_5457330.html. Accessed 21 May 2021.

[53] The Euroqol Group. EQ-5D-5L User guide: basic information on how to use the EQ-5D-5L instrument (Version 3.0). 2019. https://euroqol.org/publications/user-guides/.

[54] Luo N, Liu G, Li M (2017). Estimating an EQ-5D-5L value set for China. Value Health.

[55] Barton GR, Sach TH, Avery AJ (2008). A comparison of the performance of the EQ-5D and SF-6D for individuals aged >or= 45 years. Health Econ.

[56] Koo TK, Li MY (2016). A guideline of selecting and reporting intraclass correlation coefficients for reliability research. J Chiropr Med.

[57] Landis JR, Koch GG (1977). The measurement of observer agreement for categorical data. Biometrics.

[58] McHorney CA, Tarlov AR (1995). Individual-patient monitoring in clinical practice: are available health status surveys adequate?. Qual Life Res.

[59] Suárez L, Tay B, Abdullah F (2018). Psychometric properties of the World Health Organization WHOQOL-BREF Quality of Life assessment in Singapore. Qual Life Res.

[60] Cohen J (1992). A power primer. Psychol Bull.

[61] Sullivan GM, Feinn R (2012). Using effect size-or why the p value is not enough. J Grad Med Educ.

[62] Cohen J (1990). Statistical power analysis for the behavioral sciences. Comput Environ Urban Syst.

[63] Fayers PM, Machin D. Quality of life: assessment, analysis and interpretation. 2002.

[64] Stucki G, Liang MH, Fossel AH (1995). Relative responsiveness of condition-specific and generic health status measures in degenerative lumbar spinal stenosis. J Clin Epidemiol.

[65] Osborne RH, Hawthorne G, Lew EA (2003). Quality of life assessment in the community-dwelling elderly: validation of the Assessment of Quality of Life (AQoL) Instrument and comparison with the SF-36. J Clin Epidemiol.

[66] Zhou BF (2002). Effect of body mass index on all-cause mortality and incidence of cardiovascular diseases–report for meta-analysis of prospective studies open optimal cut-off points of body mass index in Chinese adults. Biomed Environ Sci.

[67] Yang Z, Busschbach J, Liu G (2018). EQ-5D-5L norms for the urban Chinese population in China. Health Qual Life Outcomes.

[68] Cnossen MC, Polinder S, Vos PE (2017). Comparing health-related quality of life of Dutch and Chinese patients with traumatic brain injury: do cultural differences play a role?. Health Qual Life Outcomes.

[69] Bansback N, Sun H, Guh DP, et al; OPTIMA TEAM. Impact of the recall period on measuring health utilities for acute events. Health Econ. 2008;17(12):1413–9. 10.1002/hec.1351. 10.1002/hec.1351 18404664

