# Supplementary material for: Comparison of the measurement properties of SF-6Dv2 and EQ-5D-5L in a Chinese population health survey
Source: Health Qual Life Outcomes. 2022 Jun 16;20:96. doi: 10.1186/s12955-022-02003-y (PMC9202323; doi:10.1186/s12955-022-02003-y)
Supplement: Supplementary file 1 — Additional file 1: Table A1. The convergent validity. [file 12955_2022_2003_MOESM1_ESM.pdf]

Health and Quality of Life Outcomes

Comparison of the Measurement Properties of SF-6Dv2 and EQ-5D-5L in a Chinese Population Health Survey

Supplementary material

Shitong Xie, PhD[1,2], Dingyao Wang[2,3], Jing Wu, PhD[2,3,*], Chunyu Liu[4], Wenchen Jiang[5,**]

[1] Department of Health Research Methods, Evidence, and Impact, McMaster University, Hamilton, Ontario, Canada

[2] School of Pharmaceutical Science and Technology, Tianjin University, Tianjin, China

[3] Center for Social Science Survey and Data, Tianjin University, Tianjin, China

[4] Tianjin Health Information Research Center (Tianjin Health Development Research Center), Tianjin, China

[5] Tianjin Hospital of Integrated Traditional Chinese and Western Medicine, Tianjin, China

[*] **Corresponding author:** Jing Wu, PhD, Professor, Room 209, 24th building, Tianjin University, 92th Weijin Road, Nankai District, Tianjin, China, 300072. E-mail: jingwu@tju.edu.cn

[**] **Corresponding author:** Wenchen Jiang, Tianjin Hospital of Integrated Traditional Chinese and Western Medicine, 6th Changjiang Road, Nankai District, Tianjin, China, 300100. E-mail: 13622169808@163.com

Table A1 Correlations among dimensions of the EQ-5D-5L and SF-6Dv2 (N=19,177)

SF-6Dv2	EQ-5D-5L				
	Mobility	Selfcare	Usual activity	Pain/Discomfort	Anxiety/Depression
Physical functioning	0.47*	0.37*	0.43*	0.48*	0.30*
Role limitation	0.45*	0.39*	0.44*	0.46*	0.43*
Social functioning	0.44*	0.40*	0.44*	0.43*	0.44*
Pain	0.47*	0.37*	0.44*	**0.69***	0.40*
Mental health	0.34*	0.34*	0.37*	0.41*	**0.52***
Vitality	0.37*	0.31*	0.36*	0.48*	0.40*

*p < 0.001; Bold formatting represents a strong correlation (r > 0.5).
